# Effects of Omega-3 Fatty Acid Supplementation on Glucose Control and Lipid Levels in Type 2 Diabetes: A Meta-Analysis

**DOI:** 10.1371/journal.pone.0139565

**Published:** 2015-10-02

**Authors:** Cai Chen, Xuefeng Yu, Shiying Shao

**Affiliations:** 1 Division of Endocrinology, Tongji Hospital, Huazhong University of Science & Technology, Wuhan, PR China, 430030; 2 The center for Biomedical Research, Tongji Hospital, Huazhong University of Science & Technology, Wuhan, PR China, 430030; Weill Cornell Medical College in Qatar, QATAR

## Abstract

**Background:**

Many studies assessed the impact of marine omega-3 fatty acids on glycemic homeostasis and lipid profiles in patients with type 2 diabetes (T2DM), but reported controversial results. Our goal was to systematically evaluate the effects of omega-3 on glucose control and lipid levels.

**Methods:**

Medline, Pubmed, Cochrane Library, Embase, the National Research Register, and SIGLE were searched to identify eligible randomized clinical trials (RCTs). Extracted data from RCTs were analyzed using STATA 11.0 statistical software with fixed or random effects model. Effect sizes were presented as weighted mean differences (WMD) with 95% confidence intervals (95% CI). Heterogeneity was assessed using the Chi-square test with significance level set at *p* < 0.1.

**Results:**

20 RCT trials were included into this meta-analysis. Among patients with omega-3 supplementation, triglyceride (TG) levels were significantly decreased by 0.24 mmol/L. No marked change in total cholesterol (TC), HbA1c, fasting plasma glucose, postprandial plasma glucose, BMI or body weight was observed. High ratio of EPA/DHA contributed to a greater decreasing tendency in plasma insulin, HbAc1, TC, TG, and BMI measures, although no statistical significance was identified (except TG). FPG levels were increased by 0.42 mmol/L in Asians. No evidence of publication bias was observed in this meta-analysis.

**Conclusions:**

The ratio of EPA/DHA and early intervention with omega 3 fatty acids may affect their effects on glucose control and lipid levels, which may serve as a dietary reference for clinicians or nutritionists who manage diabetic patients.

## Introduction

Type 2 diabetes (T2DM) is a metabolic disorder characterized by hyperglycemia in the context of insulin resistance and β-cell dysfunction. Its prevalence is increasing at an alarming rate worldwide [1–3]. Epidemiological and clinical trials have demonstrated that lifestyle, in particular daily diet, is of importance in the development and therapy of T2DM. That adherence to a healthy diet can improve glycemic control has been getting more and more attention of clinicians and nutritionists [4,5].

It was reported that high fish and seafood consumption could significantly reduce the incidence of T2DM in the Finnish population [6–8]. Bang et al. attributed such benefits of fish consumption to its main components, omega 3 fatty acids (in particular for eicosapentaenoic acid (C20:5n-3, EPA) and docosahexaenoic acid (C22:6n-3,DHA))—a group of homologue fatty acids belonging to polyunsaturated fatty acids (PUFAs) [7]. However, recent studies raised inverse standpoints on the benefits of administering omega-3 to diabetic patients for T2DM prevention [9–12], which may result in the underestimation the potential benefit of omega 3 in T2DM patients.

These findings trigger the investigations of omega 3 supplementation on glucose homeostasis [13–32]. Nevertheless, inconsistent conclusions still remain. These discrepancies may be attributed to differences in study designs such as trial design and duration, ethnic population of participants recruited, as well as dosage administered. Reaching clear-cut conclusions about the benefits of omega-3 administration in diabetic patients is therefore difficult, making a meta-analysis to be of significance as a dietary reference for clinicians.

According to the first related publication of systematic review in the year 2000, omega-3 had no adverse effects on glycemic control in people with diabetes [33]. The most recent systematic review was reported by Hartweg et al, in which omega 3 supplementation was found to lower the plasma level of triglyceride (TG) but have no statistical effect on glucose or insulin [34]. These previous systematic reviews were inconclusive for the diet guidance in diabetic patients. Thus, we systematically examined the randomized clinical trials (RCTs) to explore the definitive evidence on the benefit of omega-3 in patients with T2DM and to identify the appropriate dosage/compositions of omega 3 supplementation.

## Methods

### Data sources and searches

We searched the Medline, Pubmed, Cochrane Library, Embase, the National Research Register, and SIGLE (from the beginning of each database until last search of the latter in January 2015) (S1 Table) and we used Medical Subject Heading (MeSH) terms and keywords to search for records in English to identify trials involving omega-3 or n-3 or ω-3 fatty acids; docosapentaenoic acid or DPA; eicosapentaenoic or EPA; docosahexaenoic or DHA; fish oil(s)). We combined this with diabetes mellitus, type 2 diabetes or T2DM, to identify participants with T2DM. The bibliographic sections of all publications of included or excluded trials were searched manually for additional retrieval (S1 Fig).

### Study selection

Two separate investigators reviewed the titles, abstracts and keywords to determine the relevance of studies. RCTs were considered. Full articles were retrieved for further assessment if the study 1) included subjects with T2DM, 2) included omega 3 fatty acids and placebo/vegetable oil for comparison, 3) had a minimum duration of 2 weeks, 4) assessed clinical measures including HbA1c, fasting plasma glucose (FPG), postprandial plasma glucose (PPG), fasting insulin (INS), triglycerides (TG), total cholesterol (TC), BMI, or body weight (S2 Table).

Studies were excluded if 1) omega 3 supplementation was not assessed; or 2) multifactorial intervention was not included; or 3) duration of trials were less than 2 weeks; or 4) only an effect estimate was reported with no means to calculate the corresponding CI (S2 Table).

### Data extraction

To minimize bias, all data were extracted independently from the studies by two investigators. Disagreements were resolved by consensus. The data extraction form was comprised of the type of trial (parallel, cross-over, or before-after), type of omega-3 fatty acids, control supplementations, length of intervention, study setting, diabetes diagnosis, baseline characteristics of included subjects (including patient number, age, gender, race, disease duration, complications), and relevant clinical outcomes (including HbA1c, FPG, PPG, INS, TG, TC, BMI, and body weight).

### Quality assessment

Two investigators assessed quality scores of studies independently, with inconsistency settled by consensus. We adopted the criterion system developed by Jadad and Schulz [35,36], which had a possible range from 0 to 5 with 2 as a cutoff for the selection of high-quality studies.

### Statistical analysis

Extracted data from RCTs were analyzed separately to evaluate potential interactions between omega-3 and glycemic control using STATA 11.0 statistical software with fixed-effects model. A random-effects model was applied when the heterogeneity was notable (*p*<0.1). Effect sizes are presented as weighted mean differences (WMD) with 95% confidence intervals (95% CI). Heterogeneity was assessed using the Chi-square method with significance value set at *p*<0.1. Risk of publication bias was evaluated using Egger’s test with *p*-value<0.1 as significant bias [37].

Subgroup analyses were undertaken for the following variables: 1) duration of intervention (less than 8 weeks, longer than 8 weeks); 2) dose of EPA (less than 1.8g/d EPA, more than 1.8g/d EPA); 3) dose of DHA (less than 1.0g/d DHA, more than 1.0g/d DHA); 4) ratio of EPA/DHA (EPA/DHA<1.4, 1.4≤EPA/DHA≤1.5; EPA/DHA>1.5); 5) race (US/European versus Asian).

Meta-regression analysis was performed to identify the potential sources of heterogeneity between included studies. *p*-value<0.1 was set to be statistically significant. Dose of EPA and DHA, ratio of EPA/DHA, trial duration, and race were analyzed in the regression model.

Sensitivity analyses for RCTs were carried out on studies that fulfill 1) higher than 2 points on quality scale; 2) blinding (blinded studies); 3) parallel study design; 4) exclusion of any study especially on large scale (studies with a sample size larger than 100) to estimate the degree they dominate the results.

## Results

### Description of studies

**Fig 1:**
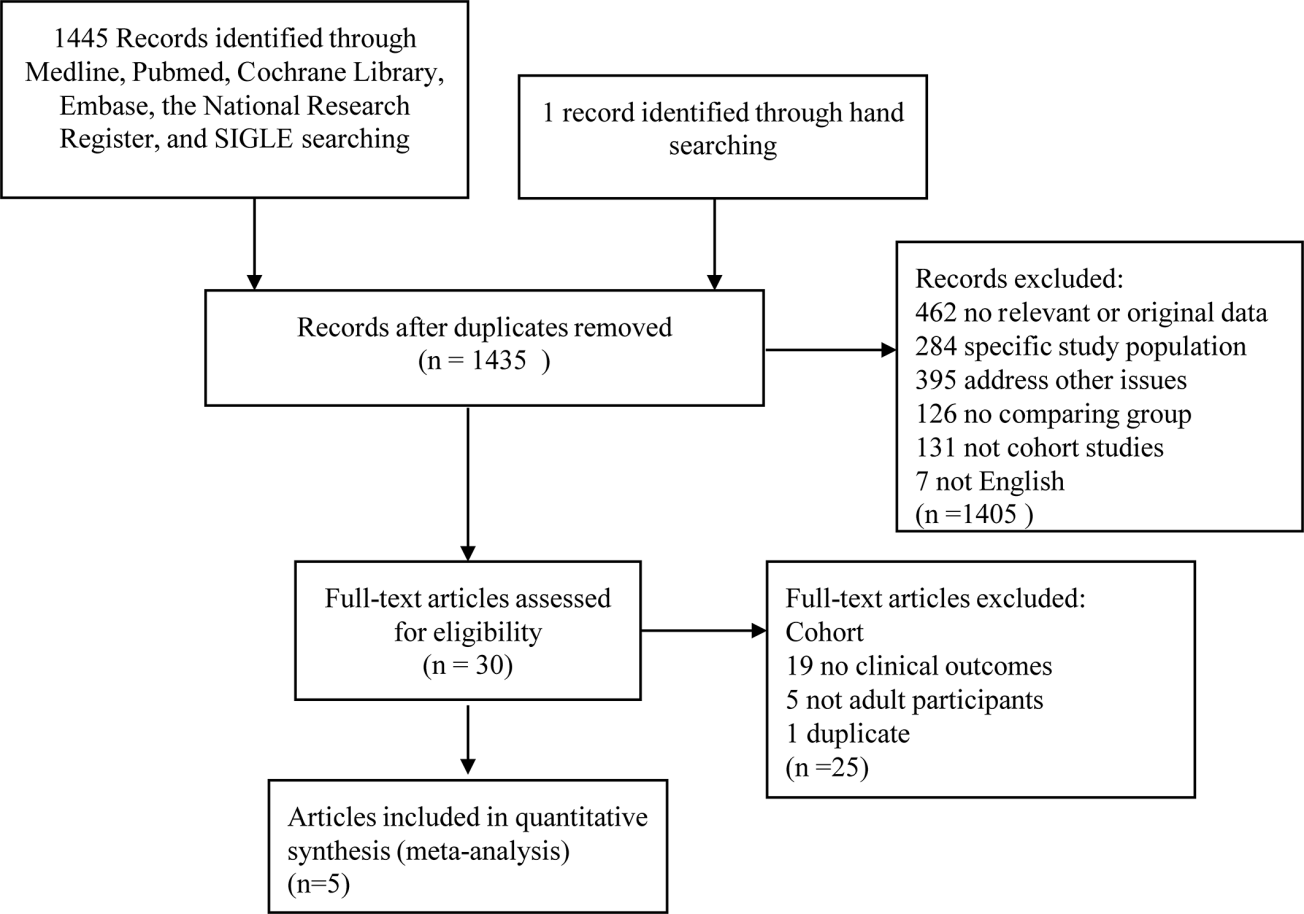
Flow chart on the articles selection process.

1445 papers were obtained from electronic searches performed in 2015 and one further trial was found from manual searching. 1324 studies were excluded for not meeting the inclusion criteria, giving a total of 111 studies for full-text articles screen. Finally, 20 RCTs were potentially eligible, which were further described below (Fig 1). The PRISMA statement is shown in S1 Dataset.

The effect of omega-3 fatty acids on glycemic control and lipid levels was the focus of 20 included RCTs. Criteria for RCTs exclusion included 24 non-randomized studies, 19 non-controlled studies, 13 publications which did not assess omega 3 supplementation, 25 studies including multifactorial intervention, 6 studies that did not include human or adult participants, and 4 trials with short duration less than 2 weeks.

The 20 RCTs included 9 parallel group design [13–21], 7 cross-over design studies [22–27,32] and 4 self-control design studies [28–31] (S3 Table). The RCTs could be classified into two categories according to the Jadad system: high quality (score > 2, n = 13), and low quality (score ≤ 2, n = 7) (S4 Table). The RCTs with high quality include 1 study scoring 5/5 [13], 5 scoring 4/5 [14,15,18,22,23] and 7 scoring 3/5 [16,17,19,20,24,25,32], which provided complete information for outcome data, adequate sequence generation, allocation concealment, and withdrawl number. All the included RCTs showed no outcome reporting bias.

A total of 1209 T2DM patients were recruited in the RCTs. The individual study sample size ranged from 6 to 414. The majority of subjects were male, aged between 51 to 64 years. Most participants had a T2DM history of 1 to 17 years, who were treated with lifestyle control or oral anti-diabetic drugs. Few of these included patients presented diabetic complications. The dose of omega-3 varied from 0.52 to 3.89g of EPA and 0 to 3.69g of DHA in T2DM patients. The dose of vegetable oil (including olive oil, safflower oil, corn oil, etc) was comparable to that of omega-3 fatty acids. In all included trials, omega-3 fatty acids were supplemented to the diet instead of a replacement for some dietary fat intake.

### Effects of interventions

**Fig 2:**
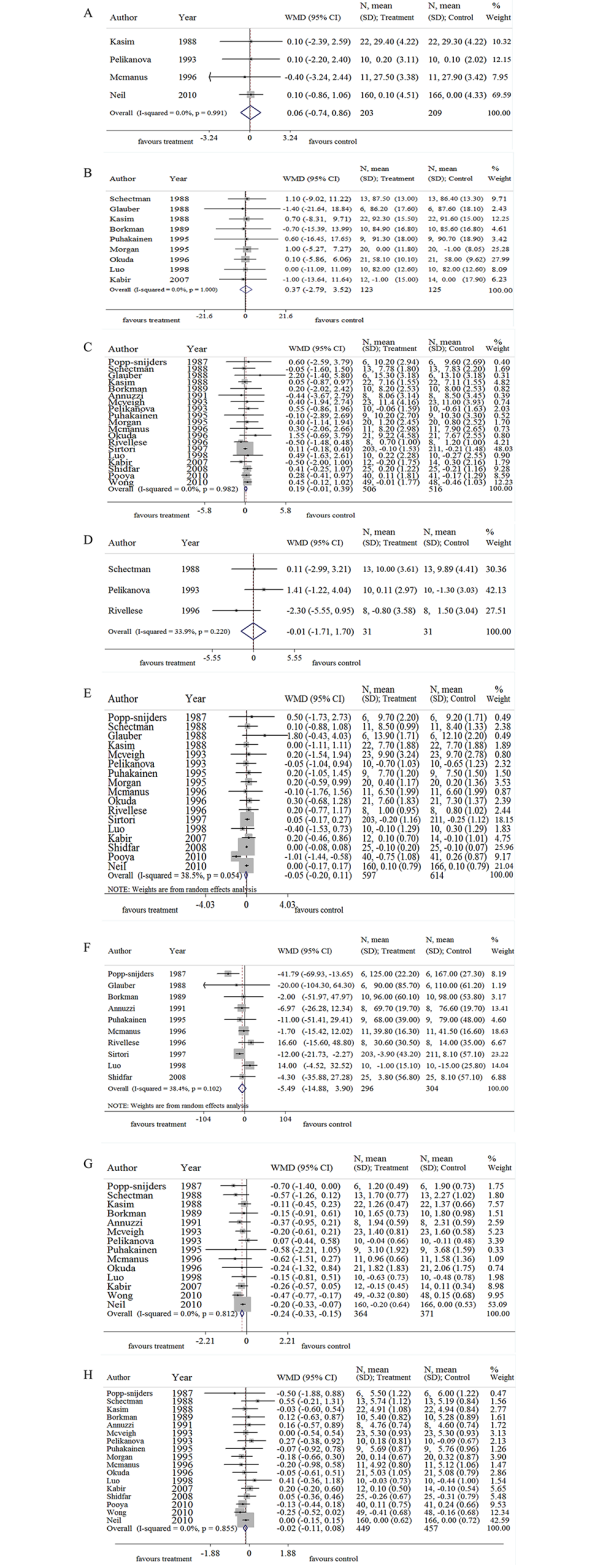
Effects of omega 3 fatty acids on BMI (A), weight (B), FPG (C), PPG (D), HbA1c (E), INS (F), TG (G), and TC (H). Note: BMI, body mass index; FPG, fast plasma glucose; PPG, postprandial glucose; HbA1c, glycosylated hemoglobin; INS, insulin; TG, Triglyceride; TC, total cholesterol; WMD, weighted mean difference.

14 of 20 RCT trials with 735 participants reported data on TG (Fig 2G). Omega 3 supplementation was related to a mean (pooled WMD) decrease of plasma TG concentration of 0.24 mmol/L (-0.33 to -0.15 mmol/L, 95% CI) compared to placebo (including vegetable oils). This decrease was remarkably significant (*p*<0.01) (Fig 2G). 17 of 20 RCT trials with 906 participants reported data on TC. But the outcome failed to show a significant decrease. Similarly, both glucose control outcomes (FPG, PPG, INS, and HbA1c) and body weight parameters (weight and BMI) with omega 3 intervention presented a non-significant difference when compared with control groups.

A moderate heterogeneity was detected in HbA1c (I-squared = 38.5, *p* = 0.054, Fig 2E). Thus, we analyzed these data according to the consumption dose of omega-3 (EPA and DHA), ratio of EPA/DHA, the trial duration, and ethnic populations.

### Subgroup analysis and heterogeneity

Table 1 shows the data from trials with high doses (more than 1.8g EPA) and low doses of EPA (less than 1.8g EPA). The levels of TG in the high dose groups were reduced by 0.3 mmol/L (-0.56 to -0.03 mmol/L, 95% CI, *p* = 0.03), similar with that in the low dose groups by 0.24mmol/L (-0.34 to -0.13 mmol/L, 95% CI, *p*<0.01). Besides plasma TG, no significant difference was identified in other clinical measures. Table 2 shows data from trials with high and low doses of DHA and Table 3 demonstrates the data from trials with different durations (Duration > 8 weeks and Duration≤8 weeks). Similar with EPA subgroups, pooled WMD was significant only in TG levels either according to DHA dosage or duration sub-grouping. Additionally, the high dose (EPA or DHA) and long duration subgroups all contributed to a greater reduction in TG levels.

**Table 1:** Omega-3 versus placebo (subgroups EPA dose).

			Overall effect			Heterogeneity	
Outcome	No. of studies	No. of participants	Statistical method	Effect size[95%CI]	P	I^2^,%	P'
Glycemic control							
FPG (mmol/L)	17	990	WMD (IV, Fixed)	0.181[-0.022,0.385]	0.081	0.0	0.964
Low dose (<1.8 g/d EPA) [13,14,16–19,22,23,30]	9	220	WMD (IV, Fixed)	0.165[-0.047,0.376]	0.127	0.0	0.819
High dose (≥1.8 g/d EPA) [20,24–29,32]	8	770	WMD (IV, Fixed)	0.396[-0.364,1.157]	0.307	0.0	0.910
PPG (mmol/L)	2	42	WMD (IV, Fixed)	-1.036[-3.280,1.208]	0.366	9.5	0.293
Low dose (<1.8 g/d EPA) [19]	1	16	WMD (IV, Fixed)	-2.300[-5.555,0.955]	0.166	―	―
High dose (≥1.8 g/d EPA) [25]	1	26	WMD (IV, Fixed)	0.110[-2.988,3.208]	0.954	―	―
HbAc1 (%)	15	1179	WMD (D+L, Random)	-0.050[-0.219,0.119]	0.565	45.7	0.028
Low dose (<1.8 g/d EPA) [14–19,22,23,30]	9	999	WMD (D+L, Random)	-0.103[-0.301,0.094]	0.305	64.1	0.004
High dose (≥1.8 g/d EPA) [20,24,25,28,29,32]	6	180	WMD (D+L, Random)	0.266[-0.187,0.719]	0.250	0.0	0.852
INS (pmol/L)	9	588	WMD (D+L, Random)	-4.288[-10.911,2.335]	0.204	2.8	0.411
Low dose (<1.8 g/d EPA) [16,18,19,22,23]	5	522	WMD (D+L, Random)	-0.448[-11.760,10.863]	0.938	49.4	0.095
High dose (≥1.8 g/d EPA) [24,26–28]	4	66	WMD (D+L, Random)	-7.572[-23.717,8.572]	0.358	0.0	0.983
Lipid parameters							
TG (mmol/L)	12	703	WMD (IV, Fixed)	-0.243[-0.339,-0.147]	<0.001	0.0	0.912
Low dose (<1.8 g/d EPA) [13,15,17,22,23,30]	6	535	WMD (IV, Fixed)	-0.235[-0.338,-0.133]	<0.001	0.0	0.543
High dose (≥1.8 g/d EPA) [24–27,29,32]	6	168	WMD (IV, Fixed)	-0.297[-0.564,-0.030]	0.029	0.0	0.950
TC(mmol/L)	15	874	WMD (IV, Fixed)	-0.023[-0.120,0.073]	0.635	0.0	0.831
Low dose (<1.8 g/d EPA) [13–17,22,23,30]	8	666	WMD (IV, Fixed)	-0.033[-0.138,0.073]	0.542	0.0	0.544
High dose (≥1.8 g/d EPA) [20,24–27,29,32]	7	208	WMD (IV, Fixed)	0.024[-0.213,0.262]	0.840	0.0	0.828
BMI (kg/m^2^)	3	392	WMD (IV, Fixed)	0.055[-0.800,0.909]	0.900	0.0	0.947
Low dose (<1.8 g/d EPA) [15,23,30]	3	392	WMD (IV, Fixed)	0.055[-0.800,0.909]	0.900	0.0	0.947
Weight(kg)	9	248	WMD (IV, Fixed)	0.365[-2.788,3.518]	0.820	0.0	1.000
Low dose (<1.8 g/d EPA) [17,22,30]	3	90	WMD (IV, Fixed)	0.088[-6.030,6.206]	0.977	0.0	0.977
High dose (≥1.8 g/d EPA) [20,24,25,27–29]	6	158	WMD (IV, Fixed)	0.466[-3.214,4.145]	0.804	0.0	1.000

Notes: BMI, body mass index; FPG, fast plasma glucose; PPG, postprandial glucose; HbA1c, glycosylated hemoglobin; INS, insulin; TG, Triglyceride; TC, total cholesterol; WMD, weighted mean difference; IV, inverse variance; D+L, DerSimonian & Laird;-, not available.

**Table 2:** Omega-3 versus placebo (subgroups DHA dosage).

			Overall effect			Heterogeneity	
Outcome	No. of studies	No. of participants	Statistical method	Effect size[95%CI]	P	I^2^,%	P'
Glycemic control							
FPG (mmol/L)	17	990	WMD (IV, Fixed)	0.181[-0.022,0.385]	0.081	0.0	0.964
Low dose (≤1.0 g/d DHA) [13,14,16–18,22,23,29]	8	752	WMD (IV, Fixed)	0.219[-0.003,0.440]	0.053	0.0	0.801
High dose (>1.0 g/d DHA) [19,20,24–28,30,32]	9	238	WMD (IV, Fixed)	-0.023[-0.538,0.493]	0.932	0.0	0.939
PPG (mmol/L)	2	42	WMD (IV, Fixed)	-1.036[-3.280,1.208]	0.366	9.5	0.293
High dose (>1.0 g/d DHA) [19,25]	2	42	WMD (IV, Fixed)	-1.036[-3.280,1.208]	0.366	9.5	0.293
HbAc1 (%)	15	1179	WMD (D+L, Random)	-0.050[-0.219,0.119]	0.565	45.7	0.028
Low dose (≤1.0 g/d DHA) [14–18,22,23,29]	8	981	WMD (D+L, Random)	-0.106[-0.313,0.101]	0.318	68.9	0.002
High dose (>1.0 g/d DHA) [19,20,24,25,28,30,32]	7	198	WMD (D+L, Random)	0.210[-0.209,0.628]	0.326	0.0	0.906
INS (pmol/L)	9	588	WMD (D+L, Random)	-4.288[-10.911,2.335]	0.204	2.8	0.411
Low dose (≤1.0 g/d DHA) [16,18,22,23]	4	506	WMD (D+L, Random)	-2.353[-13.970,9.264]	0.691	52.1	0.100
High dose (>1.0 g/d DHA) [19,24,26–28]	5	82	WMD (D+L, Random)	-2.716[-17.148,11.715]	0.712	0.0	0.755
Lipid parameters							
TG (mmol/L)	12	703	WMD (IV, Fixed)	-0.243[-0.339,-0.147]	<0.001	0.0	0.912
Low dose (≤1.0 g/d DHA) [13,15,17,22,23,29]	6	533	WMD (IV, Fixed)	-0.248[-0.355,-0.141]	<0.001	0.0	0.628
High dose (>1.0 g/d DHA) [24–27,30,32]	6	170	WMD (IV, Fixed)	-0.225[-0.438,-0.011]	0.039	0.0	0.867
TC (mmol/L)	15	874	WMD (IV, Fixed)	-0.023[-0.120,0.073]	0.635	0.0	0.831
Low dose (≤ 1.0 g/d DHA) [13–17,22,23,29]	8	664	WMD (IV, Fixed)	-0.033[-0.139,0.072]	0.533	0.0	0.543
High dose (>1.0 g/d DHA) [20,24–27,30,32]	7	210	WMD (IV, Fixed)	0.028[-0.210,0.266]	0.815	0.0	0.832
BMI (kg/m^2^)	3	392	WMD (IV, Fixed)	0.055[-0.800,0.909]	0.900	0.0	0.947
Low dose (≤ 1.0 g/d DHA) [15,23]	2	348	WMD (IV, Fixed)	0.049[-0.861,0.959]	0.916	0.0	0.744
High dose (>1.0 g/d DHA) [30]	1	44	WMD (IV, Fixed)	0.100[-2.394,2.594]	0.937	―	―
Weight(kg)	9	248	WMD (IV, Fixed)	0.365[-2.788,3.518]	0.820	0.0	1.000
Low dose (≤1.0 g/d DHA) [17,22,29]	3	88	WMD (IV, Fixed)	-0.081[-4.929,4.767]	0.974	0.0	0.988
High dose (>1.0 g/d DHA) [20,24,25,27,28,30]	6	160	WMD (IV, Fixed)	0.693[-3.459,4.844]	0.744	0.0	1.000

Notes: BMI, body mass index; FPG, fast plasma glucose; PPG, postprandial glucose; HbA1c, glycosylated hemoglobin; INS, insulin; TG, Triglyceride; TC, total cholesterol; WMD, weighted mean difference; IV, inverse variance; D+L, DerSimonian & Laird;-, not available.

**Table 3:** Omega-3 versus placebo (subgroups study duration).

			Overall effect			Heterogeneity	
Outcome	No. of studies	No. of participants	Statistical method	Effect size[95%CI]	P	I^2^,%	P'
Glycemic control							
FPG (mmol/L)	19	1022	WMD (IV, Fixed)	0.190[-0.011,0.391]	0.064	0.0	0.982
Duration > 8 weeks [13,16,18–20,23,29]	7	681	WMD (IV, Fixed)	0.190[-0.039,0.419]	0.104	0.0	0.556
Duration≤ 8 weeks [14,17,21,22,24–28,30–32]	12	341	WMD (IV, Fixed)	0.191[-0.228,0.610]	0.372	0.0	0.992
PPG (mmol/L)	3	62	WMD (IV, Fixed)	-0.005[-1.712,1.702]	0.995	33.9	0.220
Duration > 8 weeks [19]	1	16	WMD (IV, Fixed)	-2.300[-5.555,0.955]	0.166	―	―
Duration≤8 weeks [21]	2	46	WMD (IV, Fixed)	0.866[-1.139,2.870]	0.397	0.0	0.531
HbAc1 (%)	17	1211	WMD (D+L, Random)	-0.046[-0.204,0.112]	0.568	38.5	0.054
Duration >8 weeks [15,16,18–20,23,29]	7	910	WMD (D+L, Random)	0.009[-0.061,0.079]	0.799	0.0	0.988
Duration≤8 weeks [14,17,21,22,24,25,28,30–32]	10	301	WMD (D+L, Random)	-0.081[-0.555,0.394]	0.739	51.6	0.029
INS (pmol/L)	10	600	WMD (D+L, Random)	-5.489[-14.882,3.905]	0.252	38.4	0.102
Duration >8 weeks [16,18,19,23]	4	502	WMD (D+L, Random)	-5.829[-15.011,3.354]	0.213	18.5	0.298
Duration≤8 weeks [22,24,26–28,31]	6	98	WMD (D+L, Random)	-8.948[-27.854,9.957]	0.354	54.0	0.054
Lipid parameters							
TG (mmol/L)	14	735	WMD (IV, Fixed)	-0.240[-0.334,-0.147]	<0.001	0.0	0.812
Duration >8 weeks [13,15,23,29]	4	487	WMD (IV, Fixed)	-0.249[-0.365,-0.133]	<0.001	11.2	0.337
Duration≤8 weeks [17,21,22,24–27,30–32]	10	248	WMD (IV, Fixed)	-0.225[-0.382,-0.068]	0.005	0.0	0.832
TC (mmol/L)	17	906	WMD (IV, Fixed)	-0.019[-0.114,0.076]	0.690	0.0	0.855
Duration >8 weeks [13,15,16,20,23]	6	577	WMD (IV, Fixed)	-0.058[-0.172,0.057]	0.326	0.0	0.671
Duration≤8 weeks [14,17,21,22,24–27,31,32]	11	329	WMD (IV, Fixed)	0.064[-0.106,0.234]	0.459	0.0	0.841
BMI (kg/m^2^)	4	412	WMD (IV, Fixed)	0.060[-0.741,0.861]	0.883	0.0	0.991
Duration >8 weeks [15,23]	2	348	WMD (IV, Fixed)	0.049[-0.861,0.959]	0.916	0.0	0.744
Duration≤8 weeks [30] [21]	2	64	WMD (IV, Fixed)	0.100[-1.590,1.790]	0.908	0.0	1.000
Weight (kg)	9	248	WMD (IV, Fixed)	0.365[-2.788,3.518]	0.820	0.0	1.000
Duration >8 weeks [20] [29]	2	82	WMD (IV, Fixed)	0.527[-3.793,4.847]	0.811	0.0	0.838
Duration≤8 weeks [17,22,24,25,27,28,30]	7	166	WMD (IV, Fixed)	0.181[-4.432,4.794]	0.939	0.0	1.000

Notes: BMI, body mass index; FPG, fast plasma glucose; PPG, postprandial glucose; HbA1c, glycosylated hemoglobin; INS, insulin; TG, Triglyceride; TC, total cholesterol; WMD, weighted mean difference; IV, inverse variance; D+L, DerSimonian & Laird;-, not available.

Table 4 shows data from trials with high EPA/DHA ratio (EPA/DHA>1.5), intermediate ratio (1.4≤EPA/DHA≤1.5), and low ratio (EPA/DHA≤1.5). Similarly, TG levels were reduced significantly in these three subgroups with the more significant TG decrease in the high ratio subgroup (high ratio, -0.48 mmol/L, -0.736 to -0.230 mmol/L; intermediate ratio, -0.21 mmol/L, -0.384 to -0.033 mmol/L; low ratio, -0.20 mmol/L, -0.328 to -0.072 mmol/L). Additionally, a more obvious decreasing tendency was observed in HbAc1, INS, TC, and BMI measures within high ratio subgroup when compared to either low ratio or intermediate subgroups. Although no statistic significance was obtained, the downward trend of these measures was parallel to the increase of the ratios (Table 4).

**Table 4:** Omega-3 versus placebo (subgroups EPA/DHA).

			Overall effect			Heterogeneity	
Outcome	No. of studies	No. of participants	Statistical method	Effect size[95%CI]	P	I^2^,%	P'
Glycemic control							
FPG (mmol/L)	17	990	WMD (IV, Fixed)	0.181[-0.022,0.385]	0.081	0.0	0.964
1.4≤EPA/DHA≤1.5 [17,18,22,24,26–28,30,32]	9	616	WMD (IV, Fixed)	0.103[-0.160,0.366]	0.444	0.0	0.972
EPA/DHA>1.5 [13,14,23,25,29]	5	268	WMD (IV, Fixed)	0.386[-0.024,0.796]	0.065	0.0	0.829
EPA/DHA<1.4 [16,19,20]	3	106	WMD (IV, Fixed)	0.157[-0.359,0.673]	0.551	16.2	0.303
PPG (mmol/L)	2	42	WMD (IV, Fixed)	-1.036[-3.280,1.208]	0.366	9.5	0.293
EPA/DHA>1.5 [25]	1	26	WMD (IV, Fixed)	0.110[-2.988,3.208]	0.945	-	-
EPA/DHA<1.4 [19]	1	16	WMD (IV, Fixed)	-2.300[-5.555,0.955]	0.166	-	-
HbAc1 (%)	15	1179	WMD (D+L, Random)	-0.050[-0.219,0.119]	0.565	45.7	0.028
1.4≤EPA/DHA≤1.5 [17,18,22,24,28,30,32]	7	580	WMD (D+L, Random)	0.067[-0.130,0.264]	0.503	0.0	0.779
EPA/DHA>1.5 [14,23,25,29]	4	167	WMD (D+L, Random)	-0.277[-1.069,0.514]	0.492	66.7	0.029
EPA/DHA<1.4 [15,16,19,20]	4	432	WMD (D+L, Random)	0.003[-0.071,0.077]	0.938	0.0	0.939
INS (pmol/L)	9	588	WMD (IV, Fixed)	-4.568[-10.943,1.807]	0.160	2.8	0.411
1.4≤EPA/DHA≤1.5 [18,22,24,26–28]	6	500	WMD (IV, Fixed)	-6.642[-14.239,0.956]	0.087	18.2	0.295
EPA/DHA>1.5 [23]	1	22	WMD (IV, Fixed)	-1.700[-15.419,12.019]	0.808	-	-
EPA/DHA<1.4 [16,19]	2	66	WMD (IV, Fixed)	5.947[-16.599,28.493]	0.605	0.0	0.364
Lipid parameters							
TG (mmol/L)	12	703	WMD (IV, Fixed)	-0.243[-0.339,-0.147]	<0.001	0.0	0.912
1.4≤EPA/DHA≤1.5 [17,22,24,26,27,30,32]	7	190	WMD (IV, Fixed)	-0.209[-0.384,-0.033]	0.020	0.0	0.986
EPA/DHA>1.5 [13,23,25]	4	187	WMD (IV, Fixed)	-0.483[-0.736,-0.230]	<0.001	0.0	0.950
EPA/DHA<1.4 [15]	1	326	WMD (IV, Fixed)	-0.200[-0.328,-0.072]	0.002	-	-
TC (mmol/L)	15	874	WMD (IV, Fixed)	-0.023[-0.120,0.073]	0.635	0.0	0.831
1.4≤EPA/DHA≤1.5 [17,22,24,26,27,30,32]	7	190	WMD (IV, Fixed)	0.116[-0.110,0.343]	0.313	0.0	0.968
EPA/DHA>1.5 [13,14,23,25,29]	5	268	WMD (IV, Fixed)	-0.141[-0.321,0.040]	0.127	0.0	0.417
EPA/DHA<1.4 [15,16,20]	3	416	WMD (IV, Fixed)	-0.008[-0.140,0.124]	0.902	0.0	0.748
BMI (kg/m^2^)	3	392	WMD (IV, Fixed)	0.055[-0.800,0.909]	0.900	0.0	0.947
1.4≤EPA/DHA≤1.5 [30]	1	44	WMD (IV, Fixed)	0.100[-2.394,2.594]	0.937	-	-
EPA/DHA>1.5 [23]	1	22	WMD (IV, Fixed)	-0.400[-3.242,2.442]	0.783	-	-
EPA/DHA<1.4 [15]	1	326	WMD (IV, Fixed)	0.100[-0.860,1.060]	0.838	-	-
Weight (kg)	6	248	WMD (IV, Fixed)	0.365[-2.788,3.518]	0.820	0.0	1.000
1.4≤EPA/DHA≤1.5 [17,22,24,27,28,30]	2	140	WMD (IV, Fixed)	-0.060[-5.243,5.123]	0.982	0.0	1.000
EPA/DHA>1.5 [25,29]	1	68	WMD (IV, Fixed)	0.358[-4.777,5.493]	0.891	0.0	0.867
EPA/DHA< 1.4 [20]	9	40	WMD (IV, Fixed)	1.000[-5.271,7.271]	0.755	-	-

Notes: BMI, body mass index; FPG, fast plasma glucose; PPG, postprandial glucose; HbA1c, glycosylated hemoglobin; INS, insulin; TG, Triglyceride; TC, total cholesterol; WMD, weighted mean difference; IV, inverse variance; D+L, DerSimonian & Laird;-, not available.

To our surprise, FPG level was slightly elevated in Asians (0.42 mmol/L, 0.058 to 0.781 mmol/L, *p* = 0.023) but with no significant change in Western population (0.09 mmol/L, -0.154 to 0.330 mmol/L, *p* = 0.477). Due to the limitation in study number, pooled analysis failed to be performed for PPG parameter (Table 5). Moreover, Asians showed a more marked decrease in TG level when compared to subjects within US/European group. Non-significant results were found for other assessed biomarkers including plasma insulin, HbA1c, TC, BMI, and weight although ethnic population subgroups were analyzed (Table 5).

**Table 5:** Omega-3 versus placebo (subgroups Ethnicity).

			Overall effect			Heterogeneity	
Outcome	No. of studies	No. of participants	Statistical method	Effect size[95%CI]	P	I^2^,%	P'
Glycemic control							
FPG (mmol/L)	19	1022	WMD (IV, Fixed)	0.190[-0.011,0.391]	0.064	0.0	0.982
US/European [17–28,30–32]	15	752	WMD (IV, Fixed)	0.088[-0.154,0.330]	0.477	0.0	0.993
Asian [13,14,16,29]	4	270	WMD (IV, Fixed)	0.419[0.058, 0.781]	0.023	0.0	0.766
PPG (mmol/L)	3	62	WMD (IV, Fixed)	-0.005[-1.712,1.702]	0.995	33.9	0.220
HbAc1 (%)	17	1211	WMD (D+L, Random)	-0.046[-0.204,0.112]	0.568	38.5	0.054
US/European [15,17–25,28,30–32]	14	1038	WMD (D+L, Random)	0.037[-0.087,0.161]	0.561	0.0	0.991
Asian [14,16,29]	3	173	WMD (D+L, Random)	-0.287[-1.067,0.493]	0.471	90.5	0.000
INS (pmol/L)	10	600	WMD (D+L, Random)	-5.489[-14.882,3.905]	0.252	38.4	0.102
US/European [18,19,22–24,26–28,31]	9	550	WMD (D+L, Random)	-5.583[-15.887,4.722]	0.288	45.2	0.067
Asian [16]	1	50	WMD (D+L, Random)	-4.300[-35.879,27.279]	0.790	-	-
Lipid parameters							
TG (mmol/L)	14	735	WMD (IV, Fixed)	-0.240[-0.334,-0.147]	<0.001	0.0	0.812
US/European [15,17,21–27,30–32]	12	596	WMD (IV, Fixed)	-0.215[-0.313,-0.116]	<0.001	0.0	0.881
Asian [13,29]	2	139	WMD (IV, Fixed)	-0.454[-0.739,-0.169]	0.002	0.0	0.688
TC (mmol/L)	17	906	WMD (IV, Fixed)	-0.019[-0.114,0.076]	0.690	0.0	0.855
US/European [15,17,20–27,30–32]	13	636	WMD (IV, Fixed)	0.032[-0.081,0.146]	0.578	0.0	0.915
Asian [13,14,16,29]	4	270	WMD (IV, Fixed)	-0.139[-0.312,0.034]	0.116	0.0	0.644
BMI (kg/m^2^)	4	412	WMD (IV, Fixed)	0.060[-0.741,0.861]	0.883	0.0	0.991
US/European [15,21,23,30]	4	412	WMD (IV, Fixed)	0.060[-0.741,0.861]	0.883	0.0	0.991
Weight (kg)	9	248	WMD (IV, Fixed)	0.365[-2.788,3.518]	0.820	0.0	1.000
US/European [17,20,22,24,25,27,28,30]	8	206	WMD (IV, Fixed)	0.469[-3.247,4.184]	0.805	0.0	1.000
Asian [29]	1	42	WMD (IV, Fixed)	0.100[-5.859,6.059]	0.974	0.0	-

Notes: BMI, body mass index; FPG, fast plasma glucose; PPG, postprandial glucose; HbA1c, glycosylated hemoglobin; INS, insulin; TG, Triglyceride; TC, total cholesterol; WMD, weighted mean difference; IV, inverse variance; D+L, DerSimonian & Laird;-, not available.

Moderate degree of heterogeneity for HbA1c was observed in all subgroup analyses. Thus, we performed meta-regression analysis to explore the source of heterogeneity based on the following covariates: dose of EPA (*p* = 0.551) and DHA (*p* = 0.514), ratio of EPA/DHA (*p* = 0.421), trial duration (*p* = 0.415), and race (*p* = 0.134). The results revealed that no one significant factor was responsible; however, the race tended toward heterogeneity (*p* = 0.134).

### Risk of bias and sensitivity analyses

No evidence of publication bias was observed in this meta-analysis (S2 Fig). The *p*-value for each clinical measure was 0.404 for FPG, 0.274 for PPG, 0.886 for HbA1c, 0.889 for INS, 0.102 for TG, 0.377 for TC, 0.424 for BMI, 0.159 for body weight. Sensitivity analyses were shown in S5 Table. The overall effect of omega-3 intervention remained unchanged for most markers (FFG, PPG, HbA1c, TG, TC, BMI, and Body Weight) when 1) quality score of included studies were three or more; or 2) included studies were parallel design; or 3) included studies were blinded; or 4) studies with large sample size were excluded [18]. This means that the combined results for clinical outcomes listed above were stable though the specified variables changed. The outcome of insulin levels was more sensitive to study design. Including only parallel group studies resulted in a significant decrease of fasting insulin.

## Discussion

This meta-analysis pooled 20 RCTs of omega-3 supplementation with a total of 1209 T2DM patients to extend previous systematic reviews. It was found that, in the reviewed RCT studies, supplementation of omega-3 fatty acids presented a statistically significant TG decreasing effect. This effect was most remarkable in studies that gave a high dose of omega-3 and with a longer duration, which were in accordance with previous reviews [33,34]. However, omega-3 supplementation did not cause any significant change in TC, FPP, PPP, HbA1c, INS or BMI.

Interestingly, this study found that relatively high ratio of EPA/DHA contributed to a greater decreasing tendency in HbAc1, INS, TC, TG, and BMI measures. It is known that omega-3 (n-3) fatty acids include EPA, DHA, etc. Although no statistical significance was identified in these clinical outcomes (except TG), this meta-analysis study assumes the ratio and percentage of consumed mixed omega-3 fatty acids (in particular for the ratio of EPA/DHA) to be one important variable which influences the effects of omega 3 fatty acids on glucose homeostasis and lipid profiles.

Although no statistical significance but the decreasing trend identified in fasting insulin according to our analysis, animal studies supported the positive effect of omega 3 on the improvement of insulin sensitivity [38,39]. A more recent study by Molinar-Toribio et al reported that the relatively high ratio of EPA/DHA supplementation significantly decreased inflammation status and resultantly improved the insulin sensitivity in pre-diabetic rats [40], which is accordant with our speculations. The small sample size, limited study number, and short trial duration may contribute to the non-statistical effect. Additionally, there is no consensus on the optimal cutoff of the ratio of EPA/DHA. Amongst the included trials, the ratios ranged from 0.60 to 1.87 with the cut-off points 1.4 and 1.5 as the tertiles. An appropriate cut-off for this ratio is remained to be explored, which may be a helpful reference for dietary counseling: whether daily supplementation of omega-3 with high EPA/DHA ratio is necessary in T2DM patients.

Similarly, our findings from RCT trials suggest that omega-3 supplementation was unable to affect either plasma glucose levels or HbA1c except increased fasting glucose levels in Asians. Can these findings discourage the use of omega-3 in T2DM patients? Recent meta-analyses from observational studies obtained a positive conclusion that omega 3 could significantly reduce T2DM risk in Asians [12,41,42], which is contrary to our findings. The study duration may be one possible explanation for such discrepancy. Most of the included RCT trials were performed within 12 weeks, which is far shorter than those observational studies. The short duration of RCT trials may cause the null effect on glucose control. It is known that HbA1c represents an integrated measure of glycemic control over a period of approximately 12 weeks. The use of such measurements in studies of short duration may under-estimate any effects on glycemic control. Longer duration of RCT trials is required to obtain more reliable conclusion. Additionally, elevated FPG in Asians may be attributed to the included T2DM subjects in RCTs. The subjects recruited in observational studies were healthy individuals rather than diabetic patients. Thus, we assumed that early intervention with omega 3 in healthy population may be important; while the effect of omega 3 on glucose control may be decreased or eliminated when individuals have already developed to T2DM.

Our data may provide some dietary reference for clinicians or nutritionists who treat or manage patients with T2DM. The composition of mixed omega 3 fatty acids (especially the ratio of EPA/DHA) may affect their effect on glucose control and lipid levels. Additionally, early supplementation of omega 3 in healthy individuals may bring out more beneficial clinical outcomes.

## Supporting Information

S1 Fig Search strategy to identify eligible RCTs. (TIF)

S2 Fig Publication bias of included RCT studies for FPG (A), PPG (B), HbA1c (C), INS (D), TG (E), TC (F), BMI (G), and weight (H). Bias was evaluated by Egger’s test with *p*-value<0.1 as significant bias. WMD, Weighted mean difference.
(TIF)

S1 Table Web address of database used in the search. (XLSX)

S2 Table The inclusion and exclusion criteria. (XLSX)

S3 Table Characteristics of included RCT studies. (XLSX)

S4 Table Quality of included RCT studies. (XLSX)

S5 Table Sensitivity analyses. (XLSX)

S1 Dataset PRISMA Checklist. (DOC)
