# Supplementary figures and images for: Effects of Omega-3 Fatty Acid Supplementation on Glucose Control and Lipid Levels in Type 2 Diabetes: A Meta-Analysis
Source: PLoS One. 2015 Oct 2;10(10):e0139565. doi: 10.1371/journal.pone.0139565 (PMC4591987; doi:10.1371/journal.pone.0139565)

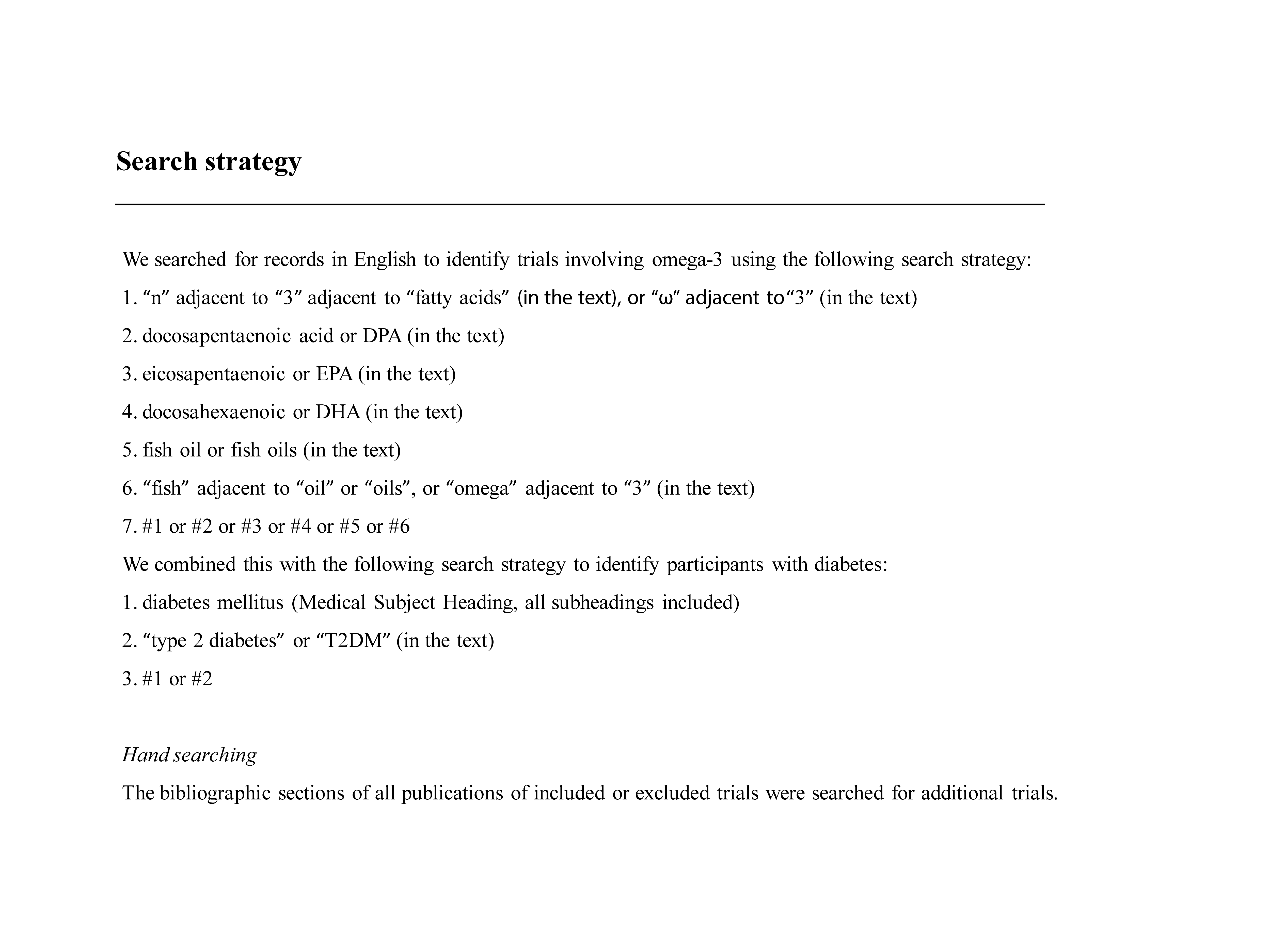

Supplement: S1 Fig — (TIF) [file pone.0139565.s001.tif]

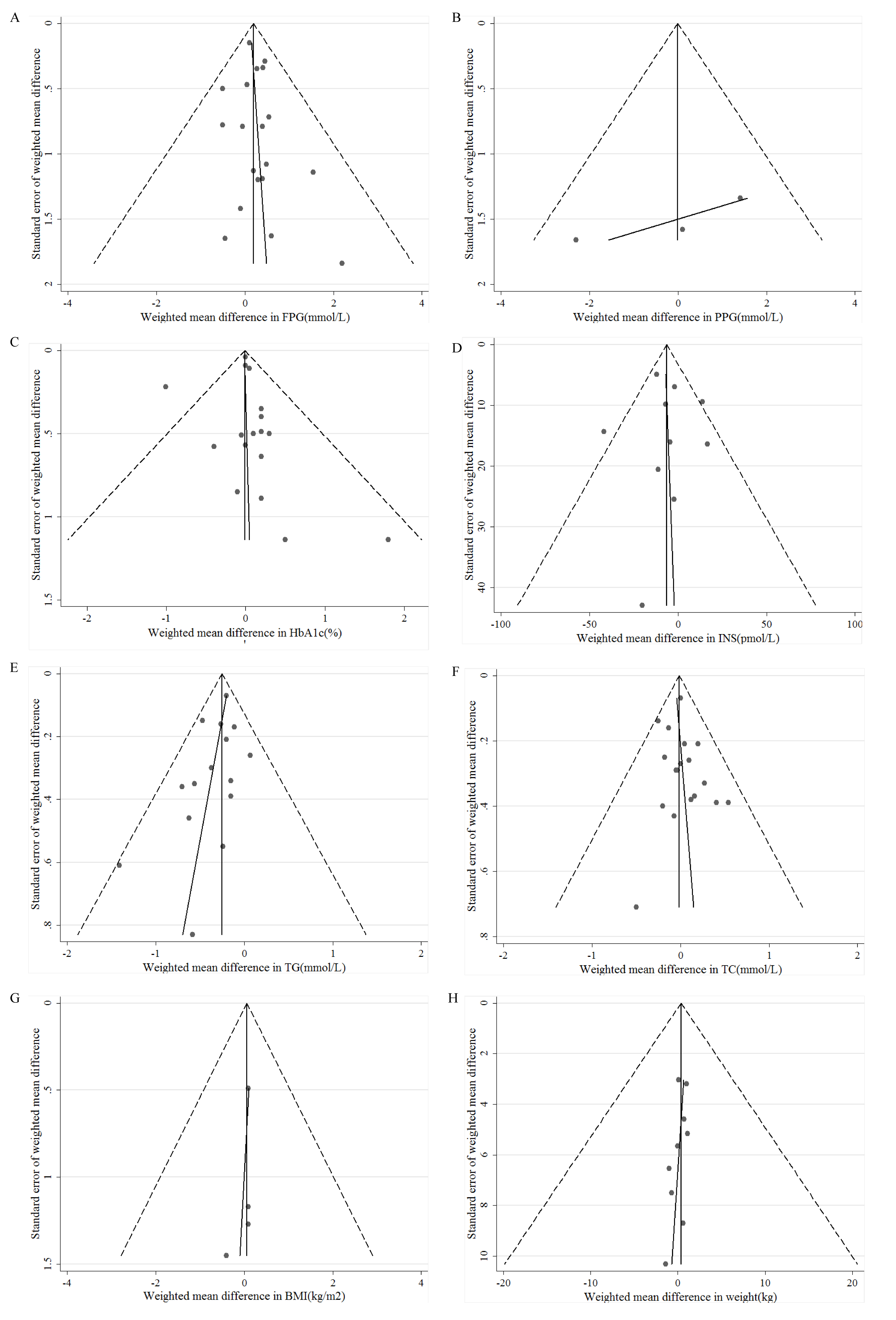

Supplement: S2 Fig — Bias was evaluated by Egger’s test with p-value<0.1 as significant bias. WMD, Weighted mean difference. (TIF) [file pone.0139565.s002.tif]
